# Supplementary material for: New Insight into Justicidin B Pathway and Production in Linum austriacum
Source: Int J Mol Sci. 2021 Mar 2;22(5):2507. doi: 10.3390/ijms22052507 (PMC7958862; doi:10.3390/ijms22052507)
Supplement: Supplementary file 1 [file ijms-22-02507-s001.zip › Supplementary Figures and Tables.pdf]

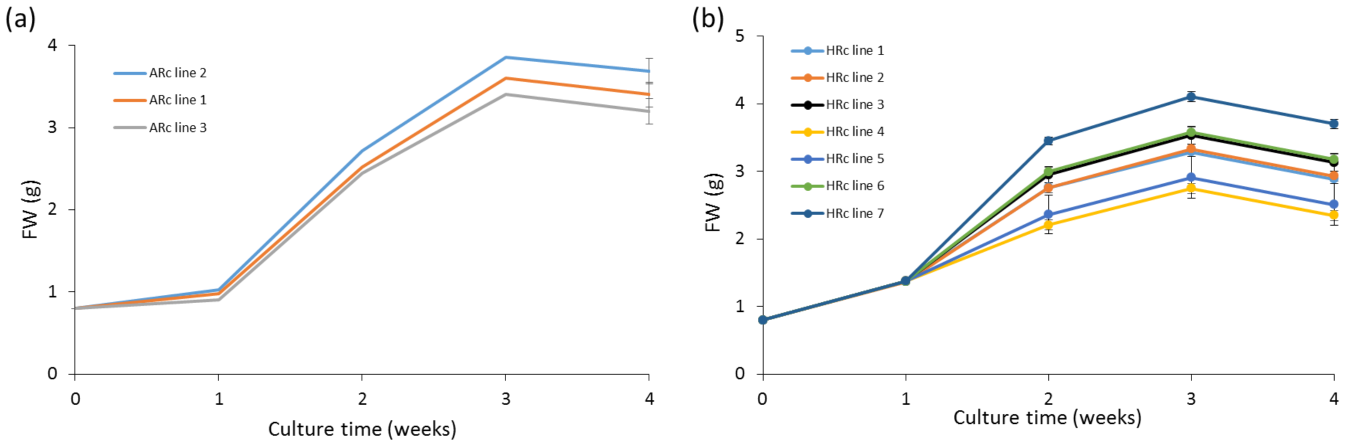

Figure S1. Growth curve of the different lines of adventitious roots (ARc) and hairy roots (HRc) obtained from *Linum austriacum* seedlings. (**a**) Growth curves of ARc lines. (**b**) Growth curves of HRc lines. FW: fresh weight expressed in g, grams. Each value represents the average of three biological replicates ± SD.

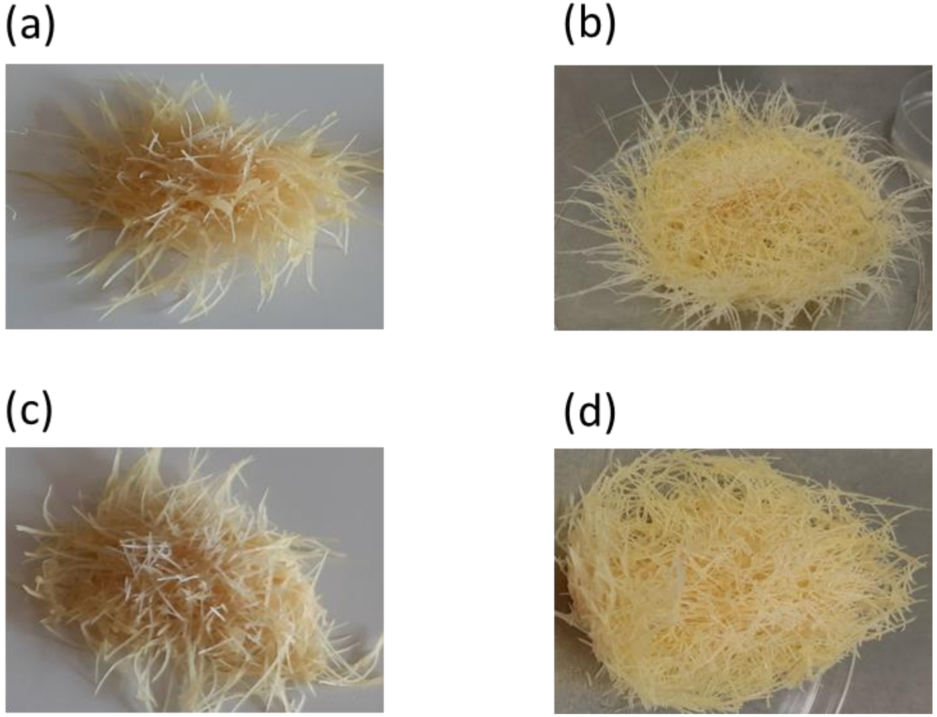

Figure S2. Picture of *Linum austriacum* adventitious (ARc) and hairy roots (HRc) tissues during the kinetic of growth. (**a**) ARc at the beginning of the growth kinetic (0 days), and (**b**) after four weeks. (**c**) HRc at the beginning of the growth kinetic (0 days), and (**d**) after four weeks.

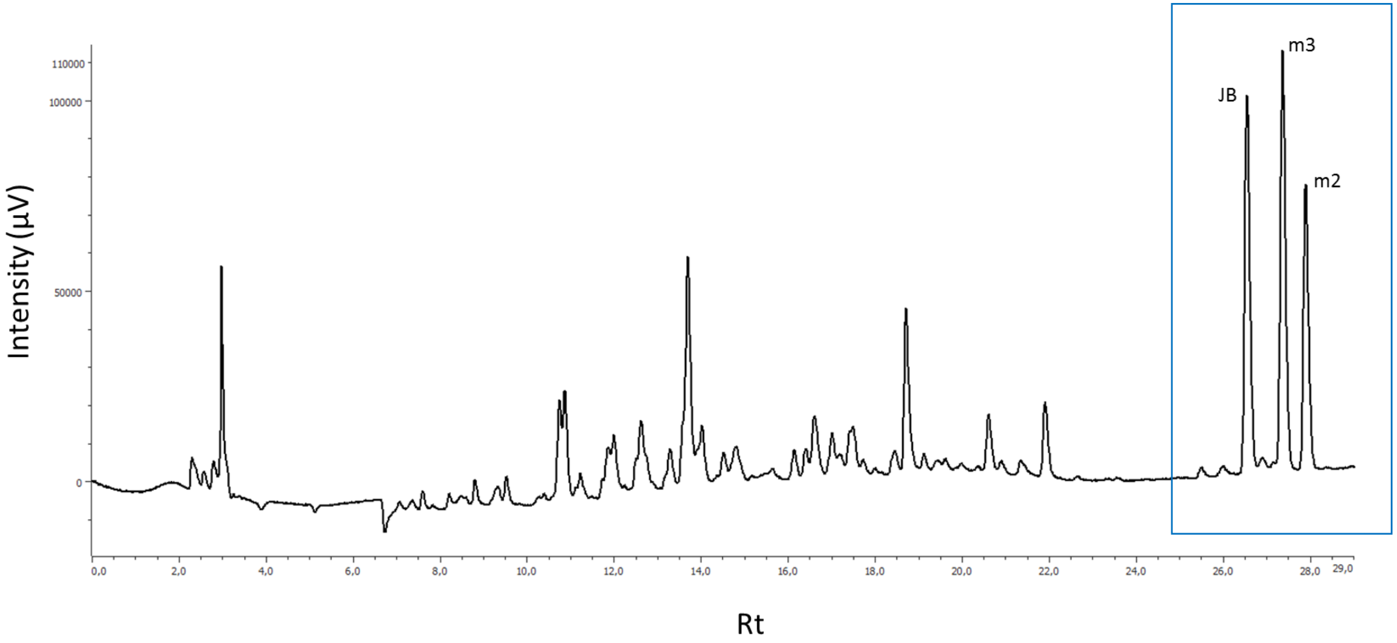

Figure S3. Full chromatogram from elicted extract of *Linum austriacum* adventitious roots (ARc). The separation was performed using a Synergi polar RP 80 Å (250 mm x 4.60 mm, 4 µm, Phenomenex) and a gradient system with water 0.1% (v/v) formic acid (A) and acetonitrile 0.1% (v/v) formic acid (B) as eluent as follows: 0-70% B for the first 26 min, from 70 to 0% B in 1 min and 0% B up to 30 min. The flow rate was 1.0 ml/min. The wavelength was monitored from 210 to 350 nm and the signals were integrated at 254 nm with a UV detector.

Table S1. Proton and carbon NMR resonance assignment (ppm) for justicidin B dissolved in $CDCl_3$. Proton and carbon chemical shift are referred to the solvent signal. Numbers are consistent with molecular structure reported in Figure 3b.

n°	^{1}H	^{13}C
1		133.2
2		128.9
3	7.13	105.9
4		151.8
5		150.3
6	7.2	106.2
7	7.72	118.4
8		139.6
9	5.4	68.18
10	6.11/6.07	101.4
11	3.83	55.9
12	4.06	56.1
1’		128.4
2’	6.87	110.8
3’		147.6
4’		147.6
5’	6.98	108.5
6’	6.85	123.5
7’		139.7
8’		118.4
C=O		170.1

Table S2. Proton and carbon NMR resonance assignment (ppm) for isojusticidin B dissolved in $CDCl_3$. Proton and carbon chemical shift are referred to the solvent signal. Numbers are consistent with the molecular structure reported in Figure 5b.

n°	^{1}H	^{13}C
1		132.8
2		128.5
3		146.2
4		150.5
5	7.5	118.2
6	7.73	124.8
7	7.82	120.9
7’		139.9
8		137.7
8’		121.6
9	5.37	67.6
10	6	101
11	3.35	60.9
12	3.97	56.8
1’		130.6
2’	6.82	109.7
3’		146.7
4’		146.5
5’	6.88	107.2
6’	6.77	121.7
C=O		169.2

Table S3. *Linum austriacum* transcripts associated to the justicidin B pathway.

	Reaction	Predicted enzymes[a]	Plant species	Enzyme	Hits identified	Hits selected
STEP 1 from L-phenylalanine to 4-coumarate	1	Lus10001405	*Linum usitatissimum*	**phenylalanine ammonia-lyase**	NS	TRINITY_DN259_c0_g1_i1
		Lus10013805 (PLA4)[c]	*Linum usitatissimum*		TRINITY_DN259_c0_g2_i1; TRINITY_DN259_c0_g3_i1; TRINITY_DN259_c0_g1_i1	TRINITY_DN259_c0_g2_i1
		Lus10023531 (PAL2)[c]	*Linum usitatissimum*		TRINITY_DN259_c0_g2_i1; TRINITY_DN259_c0_g3_i1; TRINITY_DN259_c0_g1_i1	TRINITY_DN259_c0_g3_i1
		Lus10026518 (PAL3)[c]	*Linum usitatissimum*		TRINITY_DN259_c0_g2_i1; TRINITY_DN259_c0_g3_i1; TRINITY_DN259_c0_g1_i1	
		Lus10040416 (PAL1)[c]	*Linum usitatissimum*		TRINITY_DN259_c0_g2_i1; TRINITY_DN259_c0_g3_i1; TRINITY_DN259_c0_g1_i1	
		NM_129260.3	*Arabidopsis thaliana*		TRINITY_DN259_c0_g2_i1; TRINITY_DN259_c0_g3_i1; TRINITY_DN259_c0_g1_i1	
		NM_115186.4	*Arabidopsis thaliana*		TRINITY_DN259_c0_g2_i1; TRINITY_DN259_c0_g3_i1; TRINITY_DN259_c0_g1_i1	
		NM_001203294.1	*Arabidopsis thaliana*		TRINITY_DN259_c0_g2_i1; TRINITY_DN259_c0_g3_i1; TRINITY_DN259_c0_g1_i1	
		NM_111869.4	*Arabidopsis thaliana*		TRINITY_DN259_c0_g2_i1; TRINITY_DN259_c0_g3_i1; TRINITY_DN259_c0_g1_i1	
	2	Lus10019110	*Linum usitatissimum*	***trans*-cinnamate 4-monooxygenase**	TRINITY_DN2744_c0_g2_i3	TRINITY_DN2744_c0_g1_i2
		Lus10027598	*Linum usitatissimum*		TRINITY_DN2744_c0_g1_i2	TRINITY_DN2744_c0_g2_i3
		Lus10035011	*Linum usitatissimum*		TRINITY_DN2744_c0_g1_i2;TRINITY_DN2744_c0_g2_i3	
		Lus10034449	*Linum usitatissimum*		TRINITY_DN2744_c0_g2_i3;TRINITY_DN2744_c0_g1_i2	
		Lus10021671	*Linum usitatissimum*		TRINITY_DN2744_c0_g2_i3;TRINITY_DN2744_c0_g1_i2	
		NM_128601.3	*Arabidopsis thaliana*		TRINITY_DN2744_c0_g1_i2;TRINITY_DN2744_c0_g2_i3	
STEP 2 from 4-coumarate to coniferyl alcohol 2	1	Lus10020787	*Linum usitatissimum*	**4-coumarate—CoA ligase**	NS	TRINITY_DN19496_c0_g4_i1
		Lus10024123 (CL3)[c]	*Linum usitatissimum*		TRINITY_DN19496_c0_g4_i1	TRINITY_DN19496_c0_g3_i4
		Lus10005390 (CL4)[c]	*Linum usitatissimum*		TRINITY_DN19496_c0_g4_i1	TRINITY_DN19398_c0_g2_i5
		Lus10031367	*Linum usitatissimum*		NS	TRINITY_DN19398_c0_g2_i4
		Lus10008677 (CL2)[c]	*Linum usitatissimum*		TRINITY_DN19496_c0_g4_i1	TRINITY_DN14176_c0_g1_i1
		Lus10007369	*Linum usitatissimum*		NS	TRINITY_DN19504_c0_g1_i1
		Lus10036994	*Linum usitatissimum*		NS	
		Lus10010956	*Linum usitatissimum*		NS	
		Lus10026143 (CL1)[c]	*Linum usitatissimum*		TRINITY_DN19496_c0_g4_i1	
		Lus10002791 (CL5)[c]	*Linum usitatissimum*		TRINITY_DN19398_c0_g2_i5;TRINITY_DN19398_c0_g2_i4	
		Lus10016135 (CL6)[c]	*Linum usitatissimum*		TRINITY_DN14176_c0_g1_i1	

		Lus10021431 (CL7)[c]	*Linum usitatissimum*		TRINITY_DN14176_c0_g1_i1	
		Lus10025842 (CL8)[c]	*Linum usitatissimum*		TRINITY_DN19504_c0_g1_i1	
		Lus10038259 (CL9)[c]	*Linum usitatissimum*		TRINITY_DN19504_c0_g1_i1	
		NM_104046.3	*Arabidopsis thaliana*		TRINITY_DN19496_c0_g4_i1;TRINITY_DN19496_c0_g3_i4	
		NM_105180.4	*Arabidopsis thaliana*		NS	
		NM_113019.4	*Arabidopsis thaliana*		TRINITY_DN19496_c0_g4_i1;TRINITY_DN19496_c0_g3_i4	
		AY376732.1	*Arabidopsis thaliana*		NS	
	2	Lus10027500	*Linum usitatissimum*	**4-coumarate—CoA ligase**	TRINITY_DN3440_c0_g1_i6;TRINITY_DN3440_c0_g1_i5;TRINITY_DN3440_c0_g1_i3	TRINITY_DN3440_c0_g1_i6
		Lus10041591	*Linum usitatissimum*		NS	TRINITY_DN49305_c0_g1_i1
		Lus10035519	*Linum usitatissimum*		TRINITY_DN49305_c0_g1_i1	TRINITY_DN3708_c0_g2_i5
		Lus10018983	*Linum usitatissimum*		NS	TRINITY_DN3708_c0_g1_i1
		Lus10031148	*Linum usitatissimum*		TRINITY_DN3708_c0_g2_i5	TRINITY_DN7634_c0_g4_i1
		Lus10031149	*Linum usitatissimum*		TRINITY_DN3708_c0_g1_i1	TRINITY_DN26538_c0_g1_i1
		Lus10037266	*Linum usitatissimum*		NS	TRINITY_DN3440_c0_g1_i5
		Lus10027778	*Linum usitatissimum*		NS	TRINITY_DN3440_c0_g1_i3
		Lus10028267	*Linum usitatissimum*		TRINITY_DN7634_c0_g4_i1	TRINITY_DN13078_c0_g1_i2
		Lus10026097	*Linum usitatissimum*		TRINITY_DN26538_c0_g1_i1;TRINITY_DN13078_c0_g2_i2;TRINITY_DN13078_c0_g2_i5; TRINITY_DN13078_c0_g2_i7	TRINITY_DN13078_c0_g1_i5
		Lus10026123	*Linum usitatissimum*		TRINITY_DN26538_c0_g1_i1	TRINITY_DN13078_c0_g1_i7
		Lus10040351	*Linum usitatissimum*		NS	
		Lus10039255	*Linum usitatissimum*		NS	
		Lus10039256	*Linum usitatissimum*		TRINITY_DN3440_c0_g1_i6;TRINITY_DN3440_c0_g1_i5;TRINITY_DN3440_c0_g1_i3	
		Lus10002321	*Linum usitatissimum*		TRINITY_DN26538_c0_g1_i1;TRINITY_DN13078_c0_g1_i2;TRINITY_DN13078_c0_g1_i5; TRINITY_DN13078_c0_g1_i7	
		Lus10031725	*Linum usitatissimum*		TRINITY_DN3708_c0_g2_i5	
		NM_120869.3	*Arabidopsis thaliana*		NS	
		NM_124270.4	*Arabidopsis thaliana*		TRINITY_DN26538_c0_g1_i1	
		NM_129556.4	*Arabidopsis thaliana*		NS	
		NM_114887.3	*Arabidopsis thaliana*		NS	
		AJ507825.1 [m]	*Nicotiana tabacum*		TRINITY_DN26538_c0_g1_i1	
		EF143341.1 [n]	*Coffea arabica*		TRINITY_DN26538_c0_g1_i1	
	3	Lus10033522	*Linum usitatissimum*	**p-coumaroyl ester 3'-hydroxylase**	TRINITY_DN12010_c1_g3_i1;TRINITY_DN5344_c0_g2_i1	TRINITY_DN21444_c0_g1_i2
		Lus10035844	*Linum usitatissimum*		TRINITY_DN21444_c0_g1_i2	TRINITY_DN12010_c1_g3_i1
		Lus10036627	*Linum usitatissimum*		TRINITY_DN21444_c0_g1_i2	TRINITY_DN5344_c0_g2_i1

		Lus10005780	*Linum usitatissimum*		TRINITY_DN21444_c0_g1_i2	
		Lus10020849	*Linum usitatissimum*		NS	
		NM_121034.2	*Arabidopsis thaliana*		TRINITY_DN21444_c0_g1_i2	
		NM_180006.2	*Arabidopsis thaliana*		TRINITY_DN5344_c0_g2_i1	
		DQ269126.1	*Coffea canephora*		TRINITY_DN5344_c0_g2_i1	
	**4**	Lus10040222	*Linum usitatissimum*	**caffeoyl-CoA:shikimate O-(hydroxycinnamoyl)transferase**	TRINITY_DN7634_c0_g4_i1	TRINITY_DN7634_c0_g4_i1
		Lus10024809	*Linum usitatissimum*		TRINITY_DN10576_c0_g1_i1	TRINITY_DN10576_c0_g1_i1
		AJ507825.1 [m]	*Nicotiana tabacum*		TRINITY_DN26538_c0_g1_i1	TRINITY_DN26538_c0_g1_i1
		EF143341.1 [n]	*Coffea arabica*		TRINITY_DN26538_c0_g1_i1	TRINITY_DN13078_c0_g1_i7
		NM_124270.4	*Arabidopsis thaliana*		TRINITY_DN26538_c0_g1_i1;TRINITY_DN13078_c0_g1_i7;TRINITY_DN13078_c0_g1_i5;TRINITY_DN13078_c0_g1_i2	TRINITY_DN13078_c0_g1_i2
						TRINITY_DN13078_c0_g1_i5
	**5**	Lus10009484	*Linum usitatissimum*	**caffeoyl-CoA O-methyltransferase**	TRINITY_DN15211_c1_g3_i3	TRINITY_DN15211_c1_g3_i3
		Lus10014074	*Linum usitatissimum*		TRINITY_DN11956_c0_g1_i4; TRINITY_DN11956_c0_g1_i2	TRINITY_DN11956_c0_g1_i4
		Lus10027888	*Linum usitatissimum*		TRINITY_DN11956_c0_g1_i4; TRINITY_DN11956_c0_g1_i2	TRINITY_DN11956_c0_g1_i2
		Lus10012913	*Linum usitatissimum*		TRINITY_DN11956_c0_g1_i2	TRINITY_DN59640_c0_g1_i1
		Lus10002837	*Linum usitatissimum*		TRINITY_DN11956_c0_g1_i2;TRINITY_DN11956_c0_g1_i4	
		Lus10021804	*Linum usitatissimum*		NS	
		Lus10034584	*Linum usitatissimum*		TRINITY_DN59640_c0_g1_i1	
		Lus10019841	*Linum usitatissimum*		TRINITY_DN11956_c0_g1_i4; TRINITY_DN11956_c0_g1_i2	
		Lus10010276	*Linum usitatissimum*		NS	
		Lus10036339	*Linum usitatissimum*		TRINITY_DN9212_c0_g2_i1	
		AB110168.1	*Oryza sativa*		NS	
		AF360317.1	*Arabidopsis thaliana*		TRINITY_DN11956_c0_g1_i2	
		NM_105469.2	*Arabidopsis thaliana*		TRINITY_DN11956_c0_g1_i2	
		NM_119566.5	*Arabidopsis thaliana*		NS	
	**6**	Lus10009549	*Linum usitatissimum*	**cinnamoyl-CoA reductase**	TRINITY_DN11291_c0_g4_i2	TRINITY_DN18488_c0_g1_i2
		Lus10006885 (CCR8)[c]	*Linum usitatissimum*		NS	TRINITY_DN18488_c0_g1_i1
		Lus10003780 (CCR9)[c]	*Linum usitatissimum*		NS	TRINITY_DN2679_c0_g1_i3
		Lus10008774 (CCR3)[c]	*Linum usitatissimum*		TRINITY_DN18488_c0_g1_i2;TRINITY_DN18488_c0_g1_i1	TRINITY_DN2009_c0_g1_i4
		Lus10012930 (CCR10)[c]	*Linum usitatissimum*		TRINITY_DN2679_c0_g1_i3	TRINITY_DN524_c0_g1_i1
		Lus10022239 (CCR4)[c]	*Linum usitatissimum*		TRINITY_DN18488_c0_g1_i1	TRINITY_DN3315_c0_g1_i1
		Lus10024068 (CCR2)[c]	*Linum usitatissimum*		TRINITY_DN2009_c0_g1_i4	TRINITY_DN11291_c0_g4_i2
		Lus10024138 (CCR5)[c]	*Linum usitatissimum*		TRINITY_DN2679_c0_g1_i3;TRINITY_DN3315_c0_g1_i1	

		Lus10026273 (CCR7)[c]	*Linum usitatissimum*		NS	
		Lus10030973 (CCR11)[c]	*Linum usitatissimum*		TRINITY_DN524_c0_g1_i1	
		Lus10035369 (CCR12)[c]	*Linum usitatissimum*		TRINITY_DN524_c0_g1_i1	
		Lus10041651 (CCR1)[c]	*Linum usitatissimum*		TRINITY_DN2009_c0_g1_i4	
		Lus10042399 (CCR7)[c]	*Linum usitatissimum*		TRINITY_DN524_c0_g1_i1	
		NM_001332191.1	*Arabidopsis thaliana*		TRINITY_DN524_c0_g1_i1	
		NM_119193.4	*Arabidopsis thaliana*		NS	
		NM_128919.4	*Arabidopsis thaliana*		TRINITY_DN2679_c0_g1_i3	
	7	Lus10002089 (CAD1)[b]	*Linum usitatissimum*	**coniferyl alcohol dehydrogenase**	TRINITY_DN15179_c0_g1_i2	TRINITY_DN15179_c0_g1_i2
		Lus10025706 (CAD2)[b]	*Linum usitatissimum*		TRINITY_DN3554_c0_g1_i7	TRINITY_DN3554_c0_g1_i7
		Lus10002812 (CAD3)[b,c]	*Linum usitatissimum*		TRINITY_DN15135_c0_g2_i1	TRINITY_DN15135_c0_g2_i1
		Lus10027864 (CAD4)[b,c]	*Linum usitatissimum*		TRINITY_DN15135_c0_g2_i1	TRINITY_DN15135_c0_g2_i2
		Lus10014104 (CAD5)[b,c]	*Linum usitatissimum*		TRINITY_DN15135_c0_g2_i2	TRINITY_DN848_c0_g1_i4
		Lus10019811 (CAD6)[b,c]	*Linum usitatissimum*		TRINITY_DN15135_c0_g2_i1	TRINITY_DN4458_c0_g1_i1
		Lus10009639	*Linum usitatissimum*		NS	TRINITY_DN2300_c0_g1_i3
		Lus10036147	*Linum usitatissimum*		TRINITY_DN848_c0_g1_i4	
		Lus10006507	*Linum usitatissimum*		NS	
		Lus10018119	*Linum usitatissimum*		TRINITY_DN848_c0_g1_i4	
		Lus10026290	*Linum usitatissimum*		NS	
		Lus10038445	*Linum usitatissimum*		TRINITY_DN4458_c0_g1_i1	
		Lus10008408	*Linum usitatissimum*		NS	
		Lus10041290	*Linum usitatissimum*		NS	
		Lus10023361	*Linum usitatissimum*		TRINITY_DN4458_c0_g1_i1	
		Lus10023373	*Linum usitatissimum*		NS	
		Lus10014363[c]	*Linum usitatissimum*		TRINITY_DN2300_c0_g1_i3	
		Lus10010149[c]	*Linum usitatissimum*		NS	
		Lus10026070[c]	*Linum usitatissimum*		TRINITY_DN2300_c0_g1_i3	
		Lus10023268[c]	*Linum usitatissimum*		TRINITY_DN3554_c0_g1_i7	
		Lus10035956[c]	*Linum usitatissimum*		TRINITY_DN3554_c0_g1_i7	
		Lus10002302[c]	*Linum usitatissimum*		TRINITY_DN2300_c0_g1_i3	
		Lus10002300[c]	*Linum usitatissimum*		TRINITY_DN2300_c0_g1_i3	

		Lus10009955[c]	*Linum usitatissimum*		NS	
		Lus10039595[c]	*Linum usitatissimum*		NS	
		NM_119959.4	*Arabidopsis thaliana*		TRINITY_DN3554_c0_g1_i7	
		NM_120093.5	*Arabidopsis thaliana*		TRINITY_DN15179_c0_g1_i2	
		NM_112832.4	*Arabidopsis thaliana*		TRINITY_DN15135_c0_g2_i1	
		NM_119587.4	*Arabidopsis thaliana*		TRINITY_DN15135_c0_g2_i1	
		NM_127743.4	*Arabidopsis thaliana*		TRINITY_DN15179_c0_g1_i2	
		Lus10032331 (DIR 1)[d e]	*Linum usitatissimum*		TRINITY_DN1387_c1_g2_i1	TRINITY_DN1387_c1_g2_i1
		Lus10024715 (DIR 2)[d e]	*Linum usitatissimum*		TRINITY_DN1387_c1_g2_i1	TRINITY_DN1151_c0_g1_i1
		Lus10024714 (DIR 3)[d e]	*Linum usitatissimum*		TRINITY_DN1387_c1_g2_i1	TRINITY_DN1151_c0_g1_i3
		Lus10017538 (DIR 4)[d e]	*Linum usitatissimum*		TRINITY_DN14200_c0_g2_i1	TRINITY_DN13490_c0_g2_i1
		Lus10028749 (DIR5)[d e]	*Linum usitatissimum*		TRINITY_DN15585_c0_g1_i1	TRINITY_DN14200_c0_g2_i1
		Lus10017539 (DIR6)[d e]	*Linum usitatissimum*		TRINITY_DN15585_c0_g1_i1	TRINITY_DN15585_c0_g1_i1
		Lus10005062 (DIR7)[d]	*Linum usitatissimum*		TRINITY_DN6678_c0_g1_i1	TRINITY_DN16234_c0_g6_i1
		Lus10034478 (DIR10)[d]	*Linum usitatissimum*		TRINITY_DN553_c2_g1_i1	TRINITY_DN18312_c0_g1_i1
		Lus10034482 (DIR15)[d]	*Linum usitatissimum*		TRINITY_DN2578_c1_g1_i1	TRINITY_DN18312_c0_g1_i2
		Lus10025067 (DIR16)[d]	*Linum usitatissimum*		TRINITY_DN553_c2_g2_i1	TRINITY_DN2578_c1_g1_i1
		Lus10034476 (DIR18)[d]	*Linum usitatissimum*		TRINITY_DN553_c2_g2_i1	TRINITY_DN4521_c0_g1_i1
		Lus10029306 (DIR20)[d]	*Linum usitatissimum*		TRINITY_DN1151_c0_g1_i3; TRINITY_DN1151_c0_g1_i1	TRINITY_DN4521_c0_g1_i2
**Step 3 from coniferyl alcohol to matairesinol**	**2**	Lus10016237 (DIR21)[d]	*Linum usitatissimum*	**Dirigent protein**	TRINITY_DN1151_c0_g1_i3; TRINITY_DN1151_c0_g1_i1	TRINITY_DN46372_c0_g1_i1
		Lus10042488 (DIR23)[d]	*Linum usitatissimum*		TRINITY_DN13490_c0_g2_i1	TRINITY_DN553_c2_g1_i1
		Lus10026176 (DIR24)[d]	*Linum usitatissimum*		TRINITY_DN13490_c0_g2_i1	TRINITY_DN6678_c0_g1_i1
		Lus10021084 (DIR27)[d]	*Linum usitatissimum*		TRINITY_DN4521_c0_g1_i1;TRINITY_DN4521_c0_g1_i2	
		Lus10011767 (DIR30)[d]	*Linum usitatissimum*		TRINITY_DN16234_c0_g6_i1	
		Lus10038298 (DIR34)[d]	*Linum usitatissimum*		TRINITY_DN46372_c0_g1_i1	
		Lus10017228 (DIR36)[d]	*Linum usitatissimum*		TRINITY_DN18312_c0_g1_i2	
		Lus10021082 (DIR37)[d]	*Linum usitatissimum*		TRINITY_DN18312_c0_g1_i1	
		Lus10029312 (DIR42)[d]	*Linum usitatissimum*		NS	
		AF210061.1 [i]	*Forsythia intermedia*		TRINITY_DN1387_c1_g2_i1	
		AAK38666.1 [l]	*Podophyllum peltatum*		TRINITY_DN1387_c1_g2_i1	
		ABR46205.1	*Arabidopsis thaliana*		NS	
		Glyma.05G213400.1	*Glycine max*		TRINITY_DN1387_c1_g2_i1	

	3 and 4	Lus10007599	*Linum usitatissimum*	Lariciresinol reductase /Pinoresinol/lariciresinol reductase	TRINITY_DN343_c0_g1_i3	TRINITY_DN343_c0_g1_i3
		Lus10010403	*Linum usitatissimum*		TRINITY_DN343_c0_g1_i3; TRINITY_DN6552_c0_g2_i1	TRINITY_DN343_c0_g1_i2
		Lus10012143	*Linum usitatissimum*		TRINITY_DN343_c0_g1_i3; TRINITY_DN6552_c0_g2_i1	TRINITY_DN6552_c0_g2_i1
		Lus10012147	*Linum usitatissimum*		TRINITY_DN343_c0_g1_i3; TRINITY_DN343_c0_g1_i2	
		EF050530.1[f]	*Linum perenne*		TRINITY_DN343_c0_g1_i3; TRINITY_DN6552_c0_g2_i1	
		NM_102944.3	*Arabidopsis thaliana*		TRINITY_DN343_c0_g1_i3; TRINITY_DN6552_c0_g2_i1	
		AY065214.1[o]	*Arabidopsis thaliana*		TRINITY_DN343_c0_g1_i3;TRINITY_DN6552_c0_g2_i1	
		BT002882.1[o]	*Arabidopsis thaliana*		TRINITY_DN343_c0_g1_i3;TRINITY_DN6552_c0_g2_i1	
		BT004406.1	*Arabidopsis thaliana*		TRINITY_DN343_c0_g1_i3; TRINITY_DN6552_c0_g2_i1; TRINITY_DN343_c0_g1_i2	
		U81158.1[g]	*Forsythia intermedia*		TRINITY_DN343_c0_g1_i3; TRINITY_DN343_c0_g1_i2	
	4 and 5	Lus10029368	*Linum usitatissimum*	Secoisolariciresinol dehydrogenase	TRINITY_DN6530_c0_g1_i4	TRINITY_DN6530_c0_g1_i4
		Lus10016179	*Linum usitatissimum*		TRINITY_DN6530_c0_g1_i4	TRINITY_DN8315_c0_g1_i1
		Lus10013594	*Linum usitatissimum*		NS	TRINITY_DN17210_c0_g1_i2
		Lus10021258	*Linum usitatissimum*		TRINITY_DN8315_c0_g1_i1	TRINITY_DN22425_c0_g1_i1
		AF352734.1 h	*Podophyllum peltatum*		TRINITY_DN17210_c0_g1_i2	
		AF352735.1 h	*Forsythia intermedia*		TRINITY_DN22425_c0_g1_i1	

Table S4. *Linum austriacum* transcripts up-regulated in the justicidin B pathway.

Step	Enzyme	Sequence ID	Reaction	h_2	h_5	h_24	h_96
1	phenylalanine ammonia-lyase	TRINITY_DN259_c0_g2_i1	R.1.1	1.1	0.97	0.16	0.63
		TRINITY_DN259_c0_g3_i1	R.1.2	2.68	2.16	2.7	1.48
		TRINITY_DN259_c0_g1_i1	R.1.3	1.29	0.85	1.26	0.17
	trans-cinnamate 4-monooxygenase	TRINITY_DN2744_c0_g2_i3	R.2.1	3.47	3.69	2.19	0.87
		TRINITY_DN2744_c0_g1_i2	R.2.2	2.62	2.12	1.38	1.28
2	4-coumarate—CoA ligase	TRINITY_DN19496_c0_g4_i1	R.1.1	2.74	2.27	1.92	0.87
		TRINITY_DN19496_c0_g3_i4	R.1.2	1.31	0.77	0.89	0.08
		TRINITY_DN19398_c0_g2_i4	R.1.3	1.90	-0.06	0.70	-0.58
	shikimate O-hydroxycinnamoyltransferase	TRINITY_DN13078_c0_g1_i7	R.2.2	1.96	1.66	1.53	0.71
		TRINITY_DN13078_c0_g1_i5	R.2.3	2.99	1.9	1.83	0.98
		TRINITY_DN13078_c0_g1_i2	R.2.4	2.15	1.57	1.11	0.95
		TRINITY_DN3440_c0_g1_i5	R.2.6	1.15	-1.3	-0.6	-0.16
		TRINITY_DN3440_c0_g1_i3	R.2.7	-0.03	-0.32	1.32	1.48
		TRINITY_DN7634_c0_g4_i1	R.2.8	1.12	-0.24	1.51	1.24

		TRINITY_DN49305_c0_g1_i1	R.2.9	1.52	1.53	2.12	0.14
		TRINITY_DN3708_c0_g2_i5	R.2.10	3.96	3.33	1.63	0.63
	**p-coumaroyl ester 3'-hydroxylase**	TRINITY_DN5344_c0_g2_i1	R.3.1	1.4	1.15	1.4	0.57
	**caffeoyl-CoA:shikimate O-(hydroxycinnamoyl)transferase**	TRINITY_DN10576_c0_g1_i1	R.4.1	1.45	1.15	0.3	0.25
		TRINITY_DN7634_c0_g4_i1	R.4.2	1.12	-0.24	1.51	1.24
		TRINITY_DN13078_c0_g1_i7	R.4.4	1.96	1.66	1.53	0.71
		TRINITY_DN13078_c0_g1_i5	R.4.5	2.99	1.90	1.83	0.98
		TRINITY_DN13078_c0_g1_i2	R.4.6	2.15	1.57	1.11	0.95
	**caffeoyl-CoA O-methyltransferase**	TRINITY_DN11956_c0_g1_i2	R.5.1	0.84	0.99	1.45	0.64
		TRINITY_DN11956_c0_g1_i4	R.5.2	0.91	2.31	1.12	1.06
		TRINITY_DN15211_c1_g3_i3	R.5.3	3.9	3.19	4.14	3.1
	**cinnamoyl-CoA reductase**	TRINITY_DN2009_c0_g1_i4	R.6.2	1.97	1.25	0.89	1.19
	**coniferyl alcohol dehydrogenase**	TRINITY_DN3554_c0_g1_i7	R.7.3	3.59	1.65	-1.99	-1.40
		TRINITY_DN15135_c0_g2_i1	R.7.5	0.64	0.36	1.75	1.64
**3**	**Dirigent protein**	TRINITY_DN1387_c1_g2_i1	R.2.2	4.36	3.77	5.53	3.49
		TRINITY_DN18312_c0_g1_i1	R.2.8	1.38	1.59	1.06	2.81
	**Lariciresinol reductase /Pinoresinol/lariciresinol reductase**	TRINITY_DN343_c0_g1_i3	R.3_4.1	4.89	4.34	5.69	3.52
		TRINITY_DN6552_c0_g2_i1	R.3_4.2	2.14	0.55	1.22	-1.18
		TRINITY_DN343_c0_g1_i2	R.3_4.3	4.75	4.62	5.45	2.48
	**Secoisolariciresinol dehydrogenase**	TRINITY_DN8315_c0_g1_i1	R.5_6.6	3.14	2.11	2.94	1.64
		TRINITY_DN17210_c0_g1_i2	R.5_6.7	0.58	3.38	-1.17	4.24
